# A Self-Calibrating Probabilistic Framework for 3D Environment Perception Using Monocular Vision

**DOI:** 10.3390/s20051280

**Published:** 2020-02-27

**Authors:** Razvan Itu, Radu Gabriel Danescu

**Affiliations:** Technical University of Cluj-Napoca, St. Memorandumului 28, 400114 Cluj-Napoca, Romania; razvan.itu@cs.utcluj.ro

**Keywords:** obstacle detection, measurement model, monocular vision, camera calibration

## Abstract

Cameras are sensors that are available anywhere and to everyone, and can be placed easily inside vehicles. While stereovision setups of two or more synchronized cameras have the advantage of directly extracting 3D information, a single camera can be easily set up behind the windshield (like a dashcam), or above the dashboard, usually as an internal camera of a mobile phone placed there for navigation assistance. This paper presents a framework for extracting and tracking obstacle 3D data from the surrounding environment of a vehicle in traffic, using as a sensor a generic camera. The system combines the strength of Convolutional Neural Network (CNN)-based segmentation with a generic probabilistic model of the environment, the dynamic occupancy grid. The main contributions presented in this paper are the following: A method for generating the probabilistic measurement model from monocular images, based on CNN segmentation, which takes into account the particularities, uncertainties, and limitations of monocular vision; a method for automatic calibration of the extrinsic and intrinsic parameters of the camera, without the need of user assistance; the integration of automatic calibration and measurement model generation into a scene tracking system that is able to work with any camera to perceive the obstacles in real traffic. The presented system can be easily fitted to any vehicle, working standalone or together with other sensors, to enhance the environment perception capabilities and improve the traffic safety.

## 1. Introduction

In today’s mobile and connected world, transportation and driving face multiple difficult challenges. As the roads become increasingly crowded, the energy sources become more and more insufficient, the problem of pollution becomes a world emergency, and the number of road fatalities is still too high, the industry turns towards automation to increase the transportation efficiency and safety.

Autonomous vehicles, also called intelligent vehicles, are a hot topic of research, and the manufacturers aim at completely removing the driver from behind the steering wheel, and the steering wheel completely. Such a vehicle has to have a detailed awareness of its surroundings, of its location, of the road conditions, of the traffic signs and restrictions applied in the particular area, and of the other mobile traffic participants such as vehicles, pedestrians, or bicycles. This awareness is usually achieved by employing multiple types of sensors and algorithms, and fusing their results. 

A versatile sensor for sensing the driving environment is the camera, as it can perceive both static elements of the traffic environment such as signs, lanes, and fences, and also dynamic ones such as vehicles and pedestrians. A detailed survey of environment perception for intelligent vehicles, focused mainly on camera sensors, is presented in [1].

While completely autonomous vehicles are still struggling with technical, legal, and acceptance issues, recent technological advancements in both hardware and software facilitate the development of modern driver assistance systems. These systems, based on perceiving the surroundings by means of sensors, are aimed at improving the safety of traffic participants, while making the job of the driver easier, without taking complete control of the vehicle and making the driver obsolete. The active and passive safety systems found in vehicles are based on various sensors, including cameras, lidars, or radars. The sensors can be used independently, for basic functions such as emergency braking or automatic distance keeping, or their information can be fused into a more detailed description of the environment, for a higher degree of automation of the driving process.

Sensors such as radars or lidars are called active sensors, or time-of-flight sensors. They emit electromagnetic waves in different directions, and measure the round trip time, thus measuring the distance. They are accurate, and produce 3D data that is easy to interpret and use. However, they have some disadvantages, such as reduced field-of-view (especially in the case of radar), the need to be placed outside of the vehicle for the uninterrupted path of the beam (especially for laser), reduced definition when the area covered is broad (due to spacing of the scanning rays), and the high cost. The use of such sensors requires that either the vehicle is already equipped with them from the factory, or that a professional mounts them.

Cameras are sensors that are available anywhere and to everyone, and can be placed easily inside vehicles. When two or more cameras observe the same scene, the 3D information can be directly reconstructed by means of triangulation, leading to the process of stereovision. Unfortunately, setting up a stereovision system requires rigid mounting of the cameras with respect to each other, and accurate calibration. On the other hand, a single camera can be easily set up behind the windshield (like a dashcam), or above the dashboard, usually as an internal camera of a mobile phone placed there for navigation assistance.

Computer vision algorithms designed for using a single camera are called monocular vision algorithms. A single camera produces streams of images, either monochrome or color, with a field-of-view that depends on the camera’s lens. These images can be processed either independently, or as part of a sequence. From these images, specialized algorithms can extract relevant regions and objects (segmentation), or identify the types of objects (recognition, classification). Recent developments in the field of deep learning, especially in the form of Convolutional Neural Networks (CNNs), have significantly increased the accuracy of segmentation and recognition of objects from single images, and have even made progress towards direct extraction of 3D information without the need of stereovision. However, the problem of measuring and tracking 3D objects using a single camera, a very important aspect of using the camera as a driving assistance sensor, is still not completely solved.

The 3D object detection task represents a challenge and is essential for accurate perception of the 3D world in which we live in. Acquiring 3D data is facilitated in recent years by the availability of cost effective measurement and perception sensors. 3D object detection and tracking has become increasingly popular also due to the advancement of deep learning-based techniques. 3D detection refers to obtaining information regarding the object position, orientation and size in the 3D scene. While extracting 3D object information from monocular images is a difficult task due to the limitation of the sensor, it is a goal worth pursuing due to the wide availability of such sensors and the ease of setting them up.

This paper presents a framework for extracting and tracking obstacle 3D data from the surrounding environment of a vehicle in traffic, using as sensor a generic camera. The system combines the strength of CNN-based segmentation with generic probabilistic modeling of the environment—the dynamic occupancy grid. Additional sensorial information, in the form of the observing vehicle’s speed and yaw rate, which can be acquired either from sensors equipping a smart mobile device, or from reading the CAN bus of the vehicle, is used in the process of tracking, for prediction, and in the process of automatic calibration.

The main contributions presented in this paper are the following:

- A method for generating the probabilistic measurement model from monocular images, based on CNN segmentation, which takes into account the particularities, uncertainties, and limitations of monocular vision;

- A method for automatic calibration of the extrinsic and intrinsic parameters of the camera, without the need of user assistance; and

- The integration of automatic calibration and measurement model generation into a scene tracking system that is able to work with any camera to perceive the obstacles in real traffic.

The presented system can be easily fitted to any vehicle, working standalone or together with other sensors, to enhance the environment perception capabilities and improve the traffic safety.

## 2. Related Work

Scene perception and understanding represents a major challenge for autonomous vehicles or robots. Deep learning-based techniques represent efficient tools that can aid in the process of image analysis and are used for tasks such as: object detection, classification, or semantic segmentation. Semantic segmentation of a traffic scene means interpreting the image and understanding what it contains. For this purpose, convolutional neural networks outperform traditional methods based on image processing. An important progress in the field of segmentation is presented in [2], and is based on the introduction of a CNN architecture called U-Net that features layers that encode the information and an equal number of layers for decoding it. Training this CNN for the task of segmentation requires pairs of images: the color image of the observed road scene and the labeled image where each object class is illustrated with a different color. In [3], the authors present a CNN architecture that improves prediction speed and is based on the same “encoder-decoder” style.

Neural networks can also be trained to provide inference regarding the depth map of the scene from a single image as input. This is actually achieved by training with 3D data obtained from publicly-available databases that provide stereovision information, such as the one described in [4]. Depth estimation is treated as a supervised regression problem, where a CNN is able to estimate depth from color images. In [5] the authors are able to generate the corresponding “right” image from the “left” one by training with a set of paired left-right images obtained from a stereo-camera setup. The disparity (depth) image can be generated directly by a CNN, as was initially published in [6]. A better approach is presented in [7], where the authors are able to generate the “left” image from the “right”, but also vice-versa: the “right” image from the “left” one from a stereo-camera rig. The paper also presents a cost function named “left-right consistency” that facilitates the training process. The main drawback of these methods is the fact that they do not handle well the moving objects in the scene. Still, recent methods [8] have overcome these limitations and are able to provide information regarding the speed of the ego-vehicle or even the objects in the scene.

Other methods try to use neural networks to extract 3D data for individual objects in the scene. In [9] the authors have trained a CNN that has a 2D bounding box as input, and is able to predict the 3D bounding box of the vehicles. This approach is improved in [10] by using a different cost function for regressing the local orientation. The idea is to generate bins and to regress the local object orientation as the correction needed to be applied to the center of a bin. Another idea is to extract the 3D information from the Inverse Perspective Mapping image (IPM, or bird’s-eye view). Such a method was presented in [11], where the authors map the features extracted from a color image into the bird’s-eye view image of the scene. All of these methods perform image-based 3D object detection, and usually have a lower accuracy than lidar-based methods. Paper [12] introduced an approach called “pseudo-lidar”, where the depth image features are represented similarly to lidar features. The idea is to replicate the LIDAR signal using features extracted from cameras and then to use lidar-based methods to extract the 3D information.

In order to use a camera as a sensor for environment perception, the relation between the camera pixels and the environment has to be known. The relation is established by camera calibration in the world reference frame (the world being, most of the time, relative to our own vehicle). Caltech [13] offers a calibration toolbox that is widely used in controlled environments and is based on the use of a reference pattern with a known size. However, for a vision system to be easily set up by the average user, automatic calibration is desired.

One of the most useful clues for computing automatically the position and orientation of the camera with respect to the world reference frame is the vanishing point (VP). In [14] the authors determine the vanishing point from road traffic images from a single camera by using lane lines. This method relies on a priori information regarding the camera height and lane width. This approach uses a single camera that is placed in a fixed position and where only the objects in scene are dynamic. They also rely on the flat road assumption, which is thoroughly used in the literature by other scientists. If the camera is mounted on a vehicle, then the bird’s-eye view image must be stabilized, due to the trepidations and vibrations caused by the uneven road surface. A method for stabilizing the Inverse Perspective Mapping (IPM) image is presented in [15], where the vanishing point is computed at each frame acquired from the camera. The pitch and yaw angles are determined from the VP and used to recompute the projection matrix that is used to generate the IPM image. Detecting the VP in road images can be done by using geometric properties: extracting line segments, computing their intersection, and determining the VP using a voting scheme and texture properties (extracting relevant features in images, usually with Gabor filters and analyzing their orientations [16,17], and employing voting schemes). Optical flow can also be used to calibrate cameras, as presented in [18]. The idea is to determine relevant features in consecutive frames and to extract the line segments from them. These lines will generally intersect in the vanishing point if the vehicle is travelling straight and on a flat road surface.

Environment perception systems generally use multiple sensorial input data, usually coupled with optical flow analysis. Such an approach is presented in [19] and is based on a stereovision camera setup. The dynamic vehicles from the road traffic scene are detected using the correlation between the stereo data and the feature-based optical flow. Paper [20] represents another method for vehicle detection and tracking using the fusion of stereo data and optical flow vectors. Other solutions that provide detection and tracking rely on neural networks for the detection part, as well as for the tracking. In [21], the authors provide a method for tracking using Long Short Term Memory (LSTM) neural networks, but the main disadvantage is that it heavily depends on datasets and the availability of training data. The authors mention that the tracking is trained using imagery from realistic video games (synthetic data).

## 3. Materials and Methods

**Figure 1:**
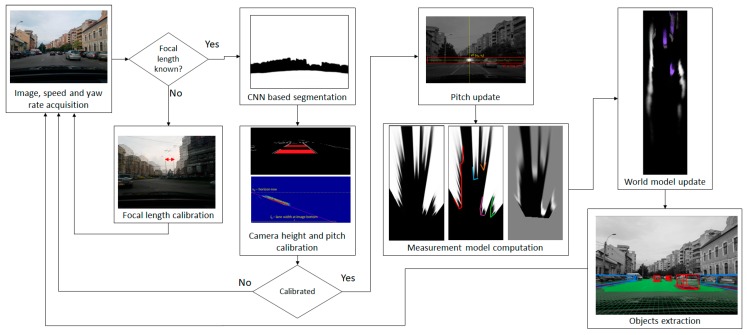
Overview of the self-calibrating obstacle detection system.

The overview of the solution is presented in Figure 1. The input data is composed of image sequences acquired from a single monocular camera, and speed and yaw rate information that can be acquired from either the vehicle’s on-board sensors, or from a mobile phone equipped with GPS and gyroscope.

In order to detect the obstacles on the road, the system has to be calibrated. The focal distance of the camera is calibrated once, by analyzing lateral displacements between consecutive frames as the vehicle rotates, knowing the rotation speed from the yaw rate sensors. After enough samples have been collected, the focal length is computed, as described in Section 3.5.

After the calibration of the focal length, the acquired images are submitted to a convolutional neural network for semantic segmentation, separating the road areas from the obstacle areas. The road areas are further processed by identifying the lane delimiter markings, which are then used for height and pitch angle calibration, as described in Section 3.5. The pitch angle is further refined by computing the image’s vanishing point, which is also used for computing the camera’s yaw angle with respect to the longitudinal axis of the host vehicle.

Using the calibrated parameters, the CNN-based segmented image is mapped in the bird’s-eye view, and the scans delimiting the road and the obstacle areas are identified and refined. Based on these scans, the probabilistic measurement model is generated. The whole process is described in Section 3.2.

The probabilistic measurement model is used to update the world model, which is detailed in Section 3.1. In the update process, the speed and yaw rate of the host vehicle are combined with the past state to generate a prediction, which is updated by the measurement, as described in Section 3.3. From the updated world model, which consists of cells occupied by dynamic particles, individual objects are extracted, as presented in Section 3.4.

### 3.1. The Probabilistic World Model

The 3D world model that surrounds a vehicle in traffic is complex and dynamic, containing obstacles of many shapes, moving in various ways. Some of the obstacles are observable (visible), and some are partly or completely occluded. A probabilistic model of the world must be able to represent all these aspects by encoding the probability of the obstacle’s existence, and the probability density of the obstacle’s speed, while allowing for efficient inference. Occupancy grids are a good compromise between descriptive power and inference efficiency. While they disregard the obstacle’s height, they allow probabilistic modeling of the existence of the obstacles (the occupancy), and the multi-hypothesis representation of the speed. Additionally, they can be easily updated by measurement data, if a suitable measurement model is used.

**Figure 2:**
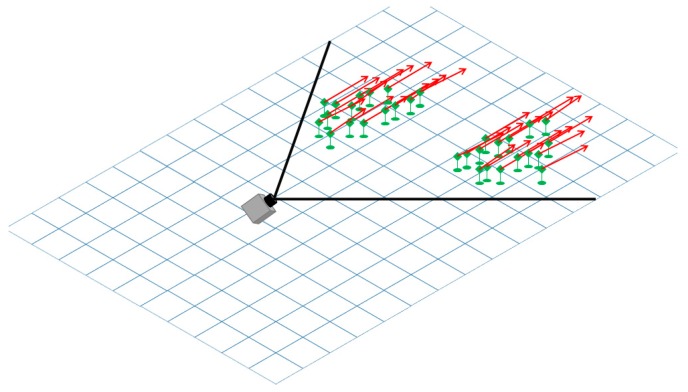
Particle-based probabilistic world model. The occupied cells in front of the camera are parts of two vehicles moving in the same direction as our own vehicle.

The particle-based dynamic occupancy grid [22,23] provides an efficient and intuitive method for representing the occupancy probability of a cell, by means of dynamic world building blocks called “particles”, as seen in Figure 2. The number of particles assigned to a grid cell is equivalent to the probability of the cell to be occupied by an obstacle, and each particle has a speed vector, allowing the population of the particles in the cell to also depict a multi-modal probability density of the obstacle’s speed. The inference mechanism follows the typical prediction–measurement–update tracking cycle, the prediction being achieved by moving particles from one cell to another based on their speed vector, and the measurement based update being achieved by multiplying, creating or removing particles based on the agreement of the prediction with the measurement data.

For the system proposed in this paper, the world is modeled as 120 × 500 cells occupancy grid, each cell representing a 20 × 20 cm area of the road, as seen in bird’s-eye view. The camera is assumed to be positioned in the middle of this grid, facing forward, meaning that half the grid is not observable directly, but can only be predicted from the observed cells. This model allows us to predict the position of obstacles when they are out of the field-of-view of the camera, and also makes the system ready to be used with an additional camera facing towards the rear.

The grid cells can hold at most *N*_C_ = 100 particles. This number is a parameter of the algorithm, controlling the accuracy of the estimation (more particles produce better estimations of occupancy and speed) at the expense of computation time. The cells that hold more than 75 particles are considered to be occupied and will be subsequently grouped into individual objects.

### 3.2. Computing the Probabilistic Measurement Model

The probabilistic measurement model is the occupancy probability derived from measurement data, used to update the world model. Formally, the measurement model is the conditional probability of the sensor measurement with respect to the world state. As the world model is based on cells and their occupancy state (occupied or free), the measurement model must provide a likelihood for the measurement related to each cell, under the assumption that the cell is either occupied or free. In order to compute these conditional probabilities, the measurement data must be mapped into the grid space.

Some sensors, such as radar, lidar or sonar, provide data that is easier to map in the world space, because their data is already 3D. A single camera provides a 2D image, containing a lot of information, but no direct mapping to the 3D space, and no direct identification of obstacle areas at sensor level (as opposed to a laser ray that will either encounter an obstacle and measure the distance towards it, or not).

**Figure 3:**
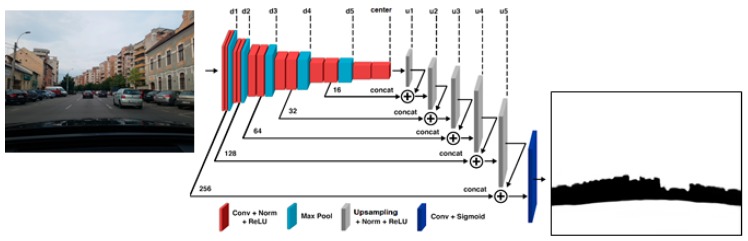
Segmenting the scene using the U-Net convolutional neuronal network.

Mapping the image information to the grid space will require first the identification of the obstacle areas using convolutional neural networks. The convolutional neural network (CNN) used in our solution is based on the U-Net architecture [2], having 5 layers for encoding the data and 5 layers for decoding. The network also features a central layer between the encoding and decoding layers. A typical encoder layer consists of the following operations: convolution using a 3 × 3 kernel, and batch normalization followed by ReLU (Rectified Linear Unit) activation. These three operations are applied again, and then they are followed by a max pooling layer (with a 2 × 2 stride). The middle layer has the same operations, minus the max pooling, whereas a typical decoder layer features up-sampling (deconvolution) with a 2 × 2 kernel, and concatenation with the homologous layer from the encoder. The decoder layer then features another deconvolution layer (with a 3 × 3 kernel), batch normalization, and ReLU activation, each applied three times. The final output of the network is given by the sigmoid activation function applied to a 1 × 1 convolution result in the last layer. Both the input and the output images have the same size, 256 × 256 pixels. Figure 3 presents a visual representation of the CNN segmentation process.

The network was trained for a total of 50 epochs using the binary cross-entropy loss function. Training is automatically stopped if there is no improvement (in this case it was stopped after 34 epochs).

For training the network, we used the state-of-the-art datasets for semantic segmentation: Cityscapes [24], KITTI [3], Berkeley Deep Drive [25], and Mapillary Vistas [26]. We used a total of more than 31,000 images, from all four datasets combined. The images with less than 2500 annotated road pixels were filtered out, resulting in 28,000 images for training and 3500 images used for validation during training. All images and labels were scaled down to 256 × 256 pixels. The main objective being to determine the drivable road area, we have only used the road class from the datasets, meaning that the image pairs used for training have the following structure: the input image (color image of the road scene, three channels) and the label image (single channel, with the road annotated as 255, and the background/non-road annotated with 0).

**Figure 4:**
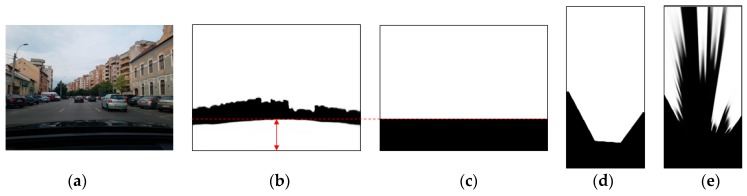
Transforming the perspective color image into usable measurement: (**a**) Perspective color image; (**b**) segmented image, highlighting the obstacle areas (white) and the free area (black); (**c**) useful area mask; (**d**) grid visibility mask; (**e**) bird’s-eye view of the segmented image.

The CNN-based semantic segmentation is able to reliably find the road and the generic obstacle areas of a color input image (Figure 4a), generating a grayscale image of the same size, with the bright regions depicting the most likely obstacle areas (Figure 4b).

These results must be mapped in the grid bird’s-eye view space, by a homography transformation that takes into account the intrinsic and extrinsic parameters of the camera with respect to the world coordinate system. The calibration of these parameters is discussed in Section 3.5. The mapping from the perspective image space to the grid space will also take into consideration the limitations of the camera’s field-of-view (the camera does not cover a rectangular area, but a cone), and the occlusion of the road in front of us by the vehicle’s hood (which is classified as obstacle, as seen from Figure 4b). The following steps are taken:

1. Find the topmost point of the hood area, as seen from Figure 4b. Using this row coordinate, we generate a mask to depict the useful area of the perspective image, as shown in Figure 4c.
2. Map the useful area mask to the grid space, using the transformation homography. Every grid area point that projects outside the perspective image, or projects in the black areas of the usefulness mask, will be set to the value 0, and all other points will be set to value 255. The result is the grid space visibility mask, shown in Figure 4d, and will be further denoted by *M*.
3. Map the segmented perspective image to the grid space, using the transformation homography. The grid cells that overlap values of zero in the visibility mask will be set to zero, and the other will be set to their corresponding segmented perspective value. The result is shown in Figure 4e, and will be further denoted by *B*.

By using visibility masks, we avoid considering the hood of our own vehicle as an obstacle. Additionally, in a later processing stage, the visibility mask will help us establish the probabilities of the grid cells to be free or occupied.

As seen from Figure 4, the bird’s-eye view mapping is only partially useful for retrieving the 3D information about the segmented obstacle areas, due to the fact that most obstacle points are not on the road and; therefore, they will not obey the assumption of zero height that is used for the homography mapping. The obstacle areas will be severely distorted, and as they approach the horizon line in the image space, they will be mapped to infinity in the bird’s-eye view space. The only useful points for measurement are, therefore, the contact points between the obstacle and the road. In the segmented bird’s-eye view image these points are the transition points between dark and bright areas. Another problem is that not even the transition points are always points of contact between obstacles and road. For example, a car touches the road with its wheels, but the space between them is above the road. Projected in the grid space, a car’s contour will present two “spikes” closer to the observer, and a gap between them.

For all the reasons described above, the grid space projection of the segmented image will undergo the processing steps described in Algorithm 1, with the aim of extracting the contours (or “scans”) of the obstacle areas as accurately as possible. We assume that the grid row coordinate is proportional to the forward distance from the camera, and the grid column coordinate is proportional to the lateral distance, and the camera is located at coordinates (*r*_cam_ = 0, *c*_cam_ = *w*_B_/2), *w*_B_ being the width of the bird’s-eye view image *B*. The threshold *T*_B_ is used to discriminate between obstacle and road areas, as the result of the CNN classification is an image of continuous grayscale values.

**Figure 5:**
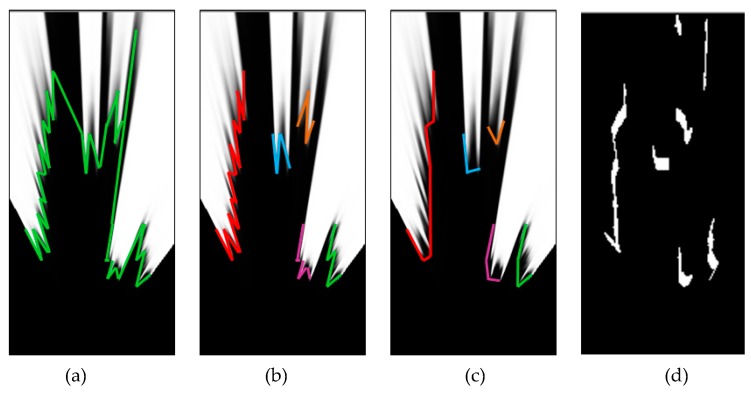
Generating the convex scans from the segmented bird’s-eye view image: (**a**) The scan lines generating by identifying transition areas along polar rays; (**b**) clustering of the scan lines; (**c**) convex hulls of the clusters; (**d**) binary image generated from extracted scans.

The first step is to transform the image into scans, an array of distances computed for each viewing angle, from 0° to 180° (the area in front of the camera). After the distance from the camera to the nearest obstacle structure is computed for every angle from 0° to 180°, only a subset of these angles will have a valid distance. For the angles that are outside of the camera’s field-of-view, and for the angles that cast rays that do not meet obstacles in the XZ range defined by the bird’s-eye view transformation, the assigned distance will remain the invalid infinity (a very large numerical value). The end points of the rays will form polygonal lines, which look like the ones in Figure 5a. The contours look fuzzy, due to the problem of incomplete contact between the obstacle and the road, and due to segmentation errors that are sometimes amplified by perspective remapping.

In order to overcome the fuzziness, we will generate convex hulls of the polylines. A danger when generating convex hulls is that it is possible to join distinct obstacles together, and “fill in” real free space. To avoid this, the polylines are split into clusters: Only adjacent rays of similar distance will be part of the same cluster. The resulted clusters are similar to the ones shown in Figure 5b. Now the convex hull can be generated by iteratively scanning groups of three rays and replacing the middle ray, if it has a higher distance than its neighbors, to the mean of the neighbors. The result is shown in Figure 5c.
**Algorithm 1**: Extraction of convex scan lines
**Input:**      bird eye view segmented image *B* **   Output:** polar distances *d*(a), for each angle *a* = 0…1801.**For** each *a* = 0…1802.  *d*(a) := *D_inf*            *// Distance for each angle, initially infinity*3.**End For**4.**For** each row *r* and column *c* of *B*  *// Compute distance to obstacle for each ray*5.  **If**
*B(r,c)*>*T*_B_6.   *a*(*r*, *c*) := atan2 (*r*-*r*_cam_, *c*-*c*_cam_) ^.^ 180/π *// angle of the ray for the obstacle cell*7.   *a*_i_ := ⌊a(r,c)+0.5⌋ 
8.   *d*_i_ := $\sqrt{{\left(r-r_{cam}\right)}^{2}+{\left(c-c_{cam}\right)}^{2}}\;$   *// distance on the ray for the cell*9.  *d*(*a*_i_) := min (*d*(*a*_i_), *d*_i_)        *// keep minimum distance for a ray*10.  **End If**11.**End For**12.**For** each *a* := 0…18013.  *K*(a) := 0         *// Cluster label for each angle a, initially 0*14.**End For**15.*N* := 0            *// Number of clusters, initially 0*16.**For***a* := 1…180        *// Cluster the rays*17.  **If**
*d*(a)<*D_inf*18.   **If** |*d*(*a*)-*d*(a-1)|<*T*_K_ and *K*(*a*-1)>0    *// Distance test*19.     *K*(*a*) := *K*(*a*-1)20.   **Else**21.     *N* := *N*+122.     *K*(*a*) := *N*23.   **End If**24.  **End If**25.**End For**26.*Changed* := true27.**While***Changed*         *// Convex hull generation, for each cluster*28.  *Changed* := false29.  **For**
*a* := 1…17930.   **If**
*d*(*a*)<*d*(*a*-1) **and**
*d*(*a*)<*d*(*a*+1) **and**
*K*(*a*)=*K*(*a*-1)=*K*(*a*+1)    *// If middle ray is longer*31.     *d*(*a*) := (*d*(*a*-1) + *d*(*a*+1))/2    *// Replace middle ray with neighbors mean*32.     *Changed* := *true*        *// Scan again*33.   **End If**34.  **End For**35.**End While**36.**Return***d*

In Algorithm 1, *B* is the input grayscale image, the remapped CNN result, and the output *d* is the distance (in grid units) for each viewing angle *a*. The clustering process uses an array *K*, which stores the cluster label for each ray of angle *a*. The number of clusters, *N*, is incremented when two adjacent rays of significant distance difference are found (the difference is compared to the clustering threshold *T*_K_, set by trial and error to an equivalent grid distance for the world distance of 3 m). Each valid ray (with a distance that is not infinite) will have a non-zero label assigned to it.

The resulted convex distances *d* can be used to generate a border image, as seen in Figure 5d. However, in order to compute the measurement model the convex ray distances themselves are more useful.

The measurement model must incorporate the measurement errors, uncertainties, and limitations. As the measurement is based on detecting the contact point between the obstacle and the road, transposed in the bird’s-eye view space, the following errors and limitations are taken into consideration:

*(1) The longitudinal errors along the observation rays*: These errors are caused by the limits of the inverse perspective mapping, or, more specific, by the uncertainty of estimating the distance to an object when knowing only its position in a perspective image. We use Equation (1) to quantify this expected error. The derivation of this equation is presented in [22].

(1) $$\sigma \left(z\right)=h\left(1+{\left(\frac{z}{h}\right)}^{2}\right)\sigma_{\alpha }+\sigma_{0},$$

In Equation (1), *h* is the camera height above the road plane, *z* is the estimated distance to the obstacle, and *σ*_α_ is the angular resolution error, which can be caused by either the limited resolution of the image, which limits the accuracy of the measurement of the vertical angle of sight for the obstacle contact point with the road surface, or by the pitching motions of the ego-vehicle. For this parameter, a value of 0.1° was chosen experimentally. By *σ*_0_ we denote an error that accounts for the non-angle related sources (imperfect segmentation, non-planar road, etc.), and this value is set, also experimentally, to 0.1 m.

*(2) The limitations of observation/visibility:* As the measurement is expressed by distances along viewing rays, there is no knowledge about the environment beyond the point where the ray reaches the obstacle. Assuming that along a ray cast at angle *a* (from 0° to 180°) we have the obstacle at distance *d*(*a*), as computed by Algorithm 1, and that an obstacle has a minimum depth *w*, we can define the occupancy probability along a ray as:

(2) $$p_{ideal}\left(a,\;z\right)=\{\begin{array}{c} 0,\;if\;z<d\left(a\right)\\ 1,\;if\;z\geq d\left(a\right)\;and\;z<d\left(a\right)+w\\ 0.5,\;if\;z\geq d\left(a\right)+w \end{array}.$$

Equation (2) states that we are certain that the cells are free before we hit the obstacle, we are certain that they are occupied for at least a small depth *w* after the obstacle is reached, and beyond that distance the probabilities of the cells being free or occupied are equal, 0.5. This equation does not take into account the sources of measurement errors, but only the occlusion caused by the first visible obstacle to the ones behind it.

In order to account for possible segmentation errors, the values 0 and 1 in Equation (2) are replaced by *p*_0_ and 1-*p*_0_, respectively, where *p*_0_ is a small value, experimentally set to 0.05. In order to account for the distance uncertainty errors, quantified by Equation (1), we define a Gaussian convolution kernel *G*(*a*), based on the standard deviation computed from Equation (1), as:

(3) $$G\left(a,i\right)=\frac{1}{\sigma \left(d\left(a\right)\right)\sqrt{2\pi }}e^{-\frac{{\left(i-i0\right)}^{2}}{2\sigma {\left(d\left(a\right)\right)}^{2}}}.$$

In Equation (3), *σ*(*d*(*a*)) is the distance standard deviation computed by Equation (1) based on the distances estimated using Algorithm 1 for each viewing angle *a*. This kernel encodes the spread of the probability of the obstacle’s existence around the estimated value, accounting for the increasing measurement uncertainty with the distance. By convolving the idealized probability *p*_ideal_(*a*) with this kernel, we obtain the realistic probability values for the obstacle’s existence along the ray of the angle *a*:

(4) $$p_{real}\left(a\right)=p_{ideal}\left(a\right)*G\left(a\right),$$

**Figure 6:**
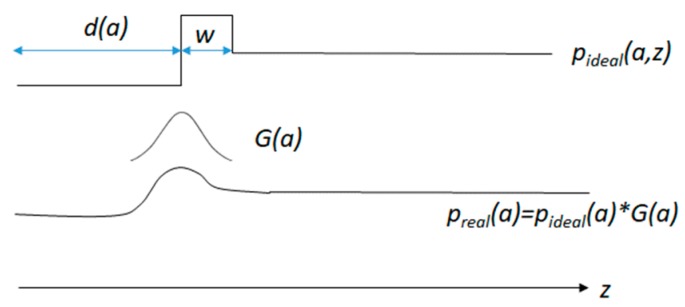
Computing the measured occupancy probability for a single ray for a given angle *a*. The obstacle is at the distance *d*(*a*), before the obstacle the area is considered free, and beyond the obstacle (of a minimum depth *w*) the state of the cells is unknown. The distance measurement error is encoded in the Gaussian kernel *G*(*a*) that will convolve the probability array.

The index *z* is omitted in Equation (4) as the convolution operation is applied to the whole array of probabilities for a given angle *a*. The steps of the measured occupancy probability computation for a single ray are presented in Figure 6.

The next step is to map these polar coordinate probabilities into the Cartesian grid space. Each row of the grid will get an assigned probability, using Algorithm 2.
**Algorithm 2**: Creation of the measurement probability grid
**Input:**  polar probabilities *p*_real_(*a*)       *visibility mask M* **Output:** measurement grid probabilities p_measured_ (*r*,*c*)1.**For** each grid row *r*2.  **For** each grid column *c*
3.   **If**
*M*(r,c)>0           *// If cell is visible, compute probability*4.     *a*_f_ := atan2 (*r*-*r*_cam_, *c* – *c*_cam_) ^.^ 180/π  *// Floating point value of the ray angle*5.     *a_0_* := $\lfloor a_{f}\rfloor \;$         *// Lower integer bound of the ray angle*6.     *a*_1_ := $\lfloor a_{f}\rfloor \;$         *// Upper integer bound of the ray angle*7.     *z*_f_ := $\sqrt{{\left(r-r_{cam}\right)}^{2}+{\left(c-c_{cam}\right)}^{2}}\;$    *// Floating point value of distance on ray*8.     *z_0_* := $\lfloor z_{f}\rfloor \;$             *// Lower integer bound of distance*9.     *z*_1_ := $\lfloor z_{f}\rfloor \;$             *// Upper integer bound of distance*10.     *p*_measured_(*r*,*c*) := LinearInterpolation(*p*_real_, *a*_0_, *a*_1_, *z*_0_, *z*_1_, *a*_f_, *z*_f_) **// 4 point interpolation**11.   **Else**12.     *p*_measured_(*r*,*c*) := 0.5    *// Cell not visible, probability is default 0.5*13.   **End if**14.  **End For**15.**End For**16.**Return***p*_measured_

In Algorithm 2, the grid is assumed to be larger than the observable scene, and the position of the camera, which is the point from where the rays are cast, is located in the grid at coordinates (*r*_cam_, *c*_cam_). In our implementation, the camera is located in the middle of the tracked grid. The row and column coordinates *r* and *c* are used to compute a distance *z*_f_ and an angle *a*_f_, as floating point values, for each observed grid cell (as indicated by the visibility mask *M*, depicted in Figure 4d). Because indexing the polar probability matrix *p*_real_(*a*,*z*) requires integer coordinates, the function LinearInterpolation is used to compute a weighted mean of the *p*_real_ values of the integer neighbors of *z*_f_ and *a*_f_. This way, we will ensure a complete and smooth coverage of the grid cells with measurement probability values.

**Figure 7:**
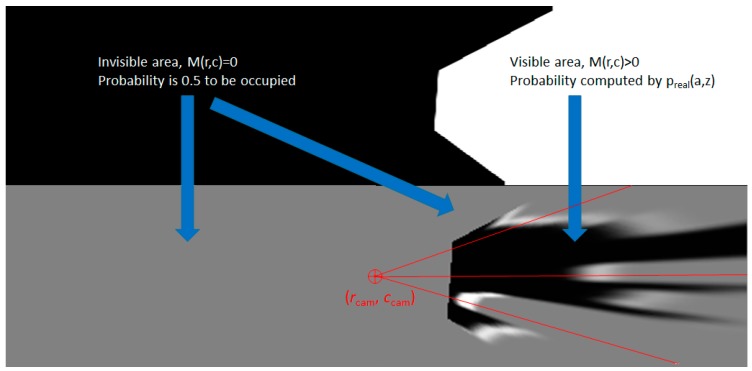
Computing the measurement occupancy probability values for all the grid cells. The cells that are not observable (*M*(r,c) = 0) have a default 0.5 value of the probability, while the other have the probability decided by the rays cast from the camera point that meet obstacle areas.

The process of generating the measurement probability grid is depicted in Figure 7. The grid area behind the camera is assumed to be invisible and; therefore, the visibility mask *M* has zero values.

### 3.3. Updating the World State

Before measurement, the probability of a grid cell at coordinates (*r*, *c*) to be occupied is given by the number of particles in that cell, particles that have been moved using the motion equations of the ego-vehicle (the forward movement expressed by the speed, and the angular movement expressed by the yaw rate), and the motion equations of the particles themselves (uniform motion based on constant speed, combined with random alterations of the position and speed), as described in [23]. We will denote this predicted probability as *p*_predicted_(*r*, *c*), the ratio between the number of predicted particles in the cell and the maximum capacity of the cell. If more particles are moved in the cell in the process of prediction, the particles in excess of the cell capacity *N*_C_ are discarded, so that always *p*_predicted_(*r*,*c*) ≤ 1.

After the measurement data is processed, each grid cell will have assigned a measured occupancy probability value *p*_measured_(*r*, *c*). The updated probability of the cell to be occupied is subsequently computed using Equation (5):

(5) $$p\left(r,c\right)=\frac{p_{\mathrm{predicted}}\left(r,c\right)p_{\mathrm{measured}}\left(r,c\right)}{p_{\mathrm{predicted}}\left(r,c\right)p_{\mathrm{measured}}\left(r,c\right)+\left(1-p_{\mathrm{predicted}}\left(r,c\right)\right)\left(1-p_{\mathrm{measured}}\left(r,c\right)\right)}.$$

As the true state of the grid is represented by its component particles, the probability computed by Equation (5) is just an intermediate step towards adjusting the particle population of each cell. In order to comply with the computed probability *p*(*r*, *c*), the number of particles in the cell must be adjusted to match the product between *p*(r, c) and *N*_C_, *N*_C_ being the maximum capacity of a cell (100 in our implementation). If the current number of particles in the cell, resulted after prediction, is lower than the target number, the particles are randomly multiplied. If the current number of particles is higher than the target, particles are randomly eliminated [23]. The random elimination or multiplication will preserve the probability distribution of the cell’s speed.

For a smoother estimation, we used an additional step before the particle multiplication/deletion process, consisting of a Gaussian smoothing of the *p*(*r*, *c*) grid.

### 3.4. Identifying Individual Objects

The updated occupancy grid is segmented into individual objects by proximity-based labeling. Clusters of occupied neighboring cells are extracted, and cuboids are fitted to them. The speeds of each cell, resulted from the speeds of individual particles within the cell, are used to estimate the speed and orientation of the resulted cuboid. If the speed of the cuboid is too low, or the standard deviation computed from the individual cell speeds is too high, the object is reported as static and no orientation is computed for it.

There are two improvements to the classical labeling algorithm that ensure a better estimation of the cuboids, and reduce the number of false positives:

- The dynamic cells are not grouped together with static cells, and also they are not grouped together with cells that have a speed that differs significantly in magnitude or orientation.
- The particles that are newly created in a cell that previously had no particles are not taken into consideration when the cell is judged to be occupied or free.

**Figure 8:**
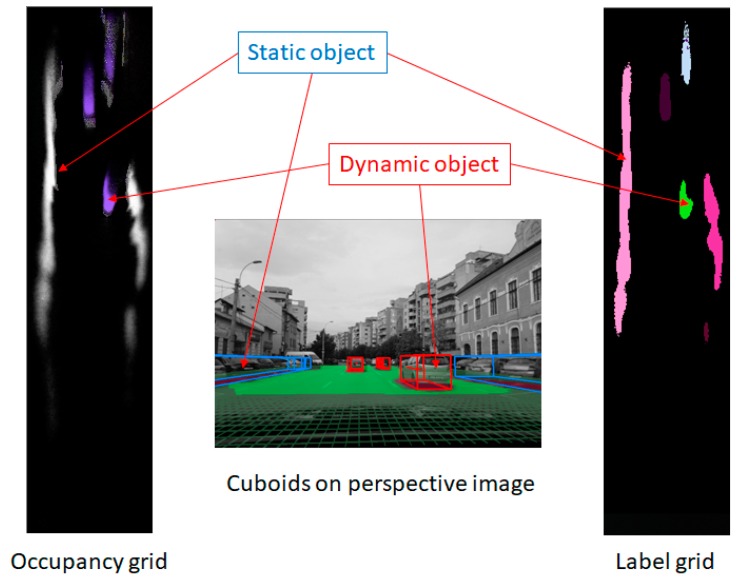
Identifying individual objects from the updated occupancy grid. The cells with high-occupancy probability are considered for clustering (labeling). The cells that have a significant average speed of their particles are considered dynamic, and will create dynamic objects, while the others will create static objects.

The process of identification of individual objects is depicted in Figure 8.

### 3.5. Automatic Camera Calibration

The proper operation of the algorithms presented in the previous sections relies on two key components: A good segmentation of the observed scene, to identify the obstacle pixels and the drivable pixels in the perspective image; and the suitable homography between the perspective image and the grid space. The segmentation can work on any image, without the need of calibration, but in order to establish the relation between the grid space and the segmentation results, the projection matrix of the camera is needed. Formally, a 3D point of coordinates (*X*_w_, *Y*_w_, *Z*_w_) is projected to the image point of coordinates (*u*, *v*) by:

(6) $$\left(\begin{array}{c} u_{s}\\ v_{s}\\ s \end{array}\right)=P\left(\begin{array}{c} X_{W}\\ Y_{W}\\ Z_{W}\\ 1 \end{array}\right).$$

The projection matrix is derived from the rotation matrix **R**_WC_, the translation vector **T**_WC_, and the intrinsic parameters matrix **A**:

(7) $$P=A[R_{WC}\;T_{WC}].$$

The rotation matrix takes into consideration the camera rotation angles pitch ( ) and yaw (φ):

(8) $$R_{WC}=\left(\begin{array}{ccc} 1 & 0 & 0\\ 0 & \cos\alpha & -\sin\alpha \\ 0 & \sin\alpha & \cos\alpha \end{array}\right).\left(\begin{array}{ccc} \cos\varphi & 0 & \sin\varphi \\ 0 & 1 & 0\\ -\sin\varphi & 0 & \cos\varphi \end{array}\right).$$

The translation vector takes into account the camera height above the road plane, *h*:

(9) $$T_{CW}=\left(\begin{array}{c} 0\\ -h\\ 0 \end{array}\right),$$

(10) $$T_{WC}=-R_{WC}T_{CW}.$$

For the intrinsic parameter matrix **A**, we will assume that the principal point is in the middle of the image (at position *H*/2, *W*/2, *H* being the image height and *W* being the image width), and the only unknown parameter is the focal length (in pixels) *f*:

(11) $$A=\left(\begin{array}{ccc} f & 0 & W/2\\ 0 & f & H/2\\ 0 & 0 & 1 \end{array}\right).$$

Our simplified intrinsic-extrinsic camera model requires the following parameters to be estimated: the focal length *f*, the camera height *h*, the pitch angle α and the yaw angle φ.

The focal length acts like a scaling factor, relating the angular displacements in the 3D world to image pixel coordinates. For example, if the camera is rotated around the vertical axis Y by a certain angle Δφ, the position of a projected world point in the image will shift on the image column axis *u* by:

(12) Δu=−fΔφ.

If the vehicle or the imaging device is equipped with a yaw rate sensor (any vehicle equipped with ESP has an on-board yaw rate sensor that can be read using the CAN bus, and most mobile phones or tables are equipped with a gyroscope which can be used to measure angular speeds), the angular difference between frames can be computed by multiplying the yaw rate with the time between frames:

(13) $$\Delta \varphi =\dot{\varphi }\Delta t.$$

The pixel displacement Δu can be measured by analyzing the displacement of image columns average brightness between frames, or can be computed by using any optical flow algorithm. Knowing the pixel displacement and the angular displacement, the focal length can be estimated:

(14) $$f=-\frac{\Delta u}{\dot{\varphi }\Delta t}.$$

Ideally, if the lateral displacement of the same feature between two consecutive frames can be determined accurately, the focal distance can be computed instantly. However, there are errors in determining all the factors involved, and many frames have an insignificant amount of rotation between them, leading to numerical instability. For these reasons, the focal length is estimated for multiple frames, the frames that have no significant yaw rate are excluded, and the final result is the median value of the list of estimated focal values. More details are presented in [27].

For the extrinsic parameters *h* (camera height) and α (pitch angle), we will analyze the distribution of a known 3D structure’s apparent width in the image space, as a function of the image line coordinate. A convenient structure that is easy to recognize and has a quasi-standard size is the lane that our vehicle travels on. The edges of the already-segmented road surface are used to find the boundaries of the current lane, by searching from the image center column towards left and right until an edge is found. This approach is very fast, and, even if it is susceptible to errors (some edges are not lane delimiters, some delimiters have no clear edges) it will provide enough valid lane width candidates to be used for calibration.

**Figure 9:**
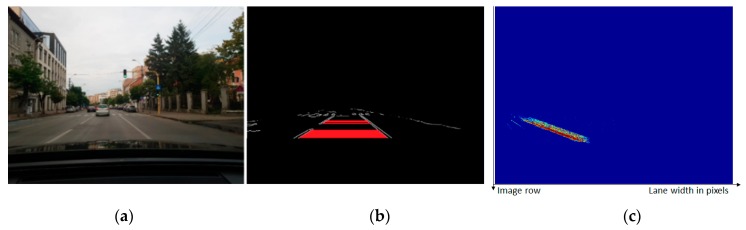
Finding the linear dependency between the lane width in the perspective image and the image row: (**a**) Perspective image, (**b**) detected lane widths, and (**c**) voting matrix counting lane width and image row pairs, shown as a heat map.

A vote matrix, of the same size as the original perspective image, was used to count each occurrence of a lane width (defined as the distance between the right and the left edge), for a certain row. Every time a lane delimiter edge pair is found, the value in the voting matrix at coordinates (row, width) is incremented. The process continues for multiple frames, so that a clear linear dependency between the row and the lane width can be established, as seen in Figure 9.

**Figure 10:**
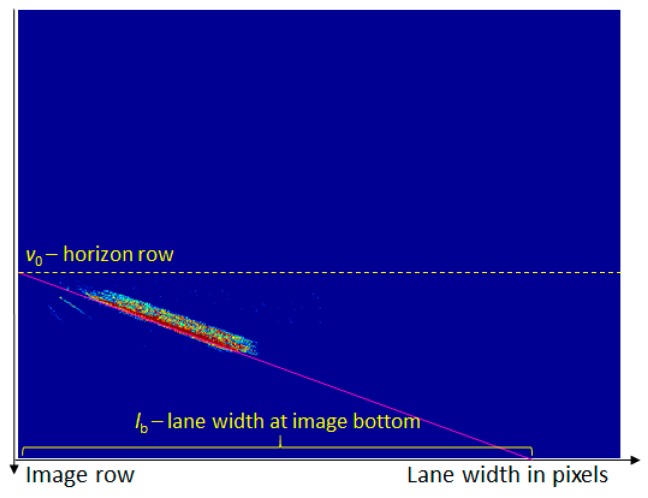
Fitting a line to the voting matrix, to establish the linear variation of lane width with the image row. The intersection of the line with the zero column is the horizon row, and the intersection with the zero row is the lane width at the bottom of the image.

The voting matrix was then analyzed by means of the Hough transform, to find the best fitting line to encode the relation between the image row and the lane width (Figure 10). In the Hough accumulator, each pixel of the (row, width) voting matrix is weighted by its value (the number of examples of the same row and width that have been encountered).

The row coordinate where the linear width distribution intersects the 0 column is the horizon row (see Figure 10), where all road structures become point-like. This row is also the geometric locus of the vanishing point. We will denote this coordinate by *v*_0_. Knowing *v*_0_ and the focal length *f*, we can estimate the pitch angle of the camera:

(15) $$\tan\alpha =\frac{\frac{H}{2}-v_{0}}{f}.$$

The intersection of the lane width line with the 0 row is the expected lane width at the bottom row of the image (see Figure 10). This width, denoted by *l*_b_, depends on the camera height above the road plane. Thus, we can compute the camera height as:

(16) $$h=D\cos\left(\frac{\pi }{2}-\alpha -\theta \right),$$

where *α* is the already computed pitch angle, *θ* is half of the vertical angular field-of-view of the camera (depends on the focal length and the image height), and *D* can be computed as:

(17) $$\;D=\frac{\sqrt{f^{2}+{\left(H/2\right)}^{2}}L}{l_{b}},\;$$

where *L* is a standard lane width for the region/city.

The yaw angle is found by detecting the vanishing point in the perspective image. The vanishing point is the point where the parallel 3D road lines meet in the image space, and is located around the already estimated horizon row. The vanishing point is found by voting along gradient direction of the road edges. As the edges converge in the vanishing point, the votes will create a maximum in the voting space, which is restricted around the *v*_0_ row.

**Figure 11:**
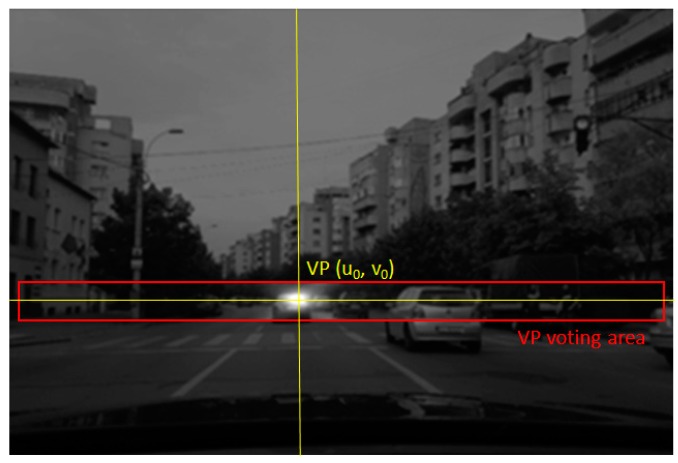
Finding the vanishing point (VP). The road edges will vote in the area around the already estimated horizon row, and the position with the maximum votes will be selected as the vanishing point.

The process of finding the vanishing point, of coordinates (*u*_0_, *v*_0_), is depicted in Figure 11.

From the estimated vanishing point, the yaw angle φ can be computed:

(18) $$\tan\varphi =\frac{\frac{W}{2}-u_{0}}{f}.$$

At this point all the required parameters can be computed. Parameters such as the focal length, the height of the camera above the road plane, and the yaw angle are static, and once they are calibrated they can be used in the obstacle detection process. However, one has to ensure that they are estimated based on sufficiently representative image data; therefore, enough frames have to be analyzed for estimating the focal distance and for generating the row vs. width voting space. For example, the focal distance calibration requires the vehicle to make turns, so driving for minutes in a straight line will not be enough, and calibration of height and pitch requires enough lanes, seen in different positions in the image space, so that the voting space in Figure 9 will show a clear linear dependency. Calibrating the yaw angle from the vanishing point detection also requires multiple results, to filter out noise and also to exclude the scenarios where our vehicle changes lanes or makes turns, and therefore the direction pointed by the road edges will not coincide with the direction of our vehicle’s heading. Thus, for measuring the yaw angle Equation (18) is applied for multiple frames, and a median value of the result is chosen.

A parameter that is constantly changing is the pitch angle, either due to the road sloping upward or downward, or due to imperfections in the road or to our maneuvers of acceleration or braking, which make the vehicle’s body oscillate. The amplitude of the pitch angle change can be more than 1°; therefore, corrections have to be constantly applied. If we denote by *v*_0_ the row coordinate of the horizon line obtained from lane width analysis (the “static” horizon), and by *v*_i_ the row coordinate of the vanishing point obtained at frame *i*, we can compute a pitch correction angle $\Delta \alpha_{i}$ for each frame:

(19) $$\Delta \alpha_{i}=\frac{v_{0}-v_{i}}{f}.$$

More details about the calibration process can be found in [27].

## 4. Evaluation and Performance

### 4.1. Data Acquisition

The development and testing process of the algorithms was based mostly on data acquired by our own recording application, written in Java and deployed on Android powered mobile devices. The application was able to interface with the following resources of the mobile phone: camera, accelerometer, gyroscope, geo-magnetic sensor, and position sensor (GPS or GLONASS depending on the used device).

**Figure 12:**
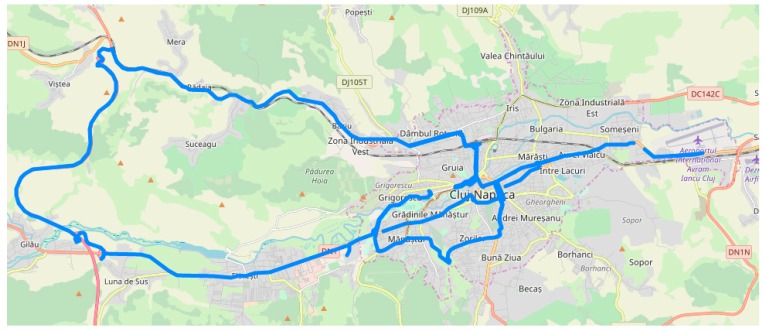
The GPS traces for all the trips recorded in Cluj-Napoca, Romania, and the near surroundings.

The mobile device’s main camera was used for acquiring images of the traffic scene. We used the main camera that is usually placed in the back of the device due to its better sensor and resolution. The other sensorial data was acquired using the available Android API’s and the data is saved on the internal storage in a text file using the current timestamp as the name. The same name was used when saving the current image/frame from the camera. Therefore, the sequences can be analyzed both locally on the device and also offline, on better hardware during the development of the algorithms. We acquired a total of over 115,200 images grouped into 27 sequences in various weather conditions, during different moments of the day and, in the city, rural road and highway scenarios. A figure containing all the GPS traces from the recorded trips in our home city and its surroundings is illustrated in Figure 12.

The dataset containing the recorded trips is publicly available at [28].

Besides our acquired sequences, publicly-available datasets were used for testing individual modules of the obstacle detection framework.

### 4.2. Segmentation Results

The CNN-based segmentation was evaluated using the validation set from CityScapes [24]. The Intersect Over Union (IoU) score that we obtained is 0.91 and the state-of-the-art [29] from Google achieves 0.98, as seen in Table 1. The result of our method is thresholded with a fixed value, we do not post-process or refine the segmentation mask.

The evaluation is done using a network trained to predict a single class (road only). We found that by training for multiple classes the score can increase (0.92 vs. 0.91). Another aspect is that the current state-of-the-art methods rely on training to predict for the full 19 classes from the dataset, and they also rely on bigger input images both for prediction and training. We chose to favor prediction speed by using smaller input images.

**Figure 13:**
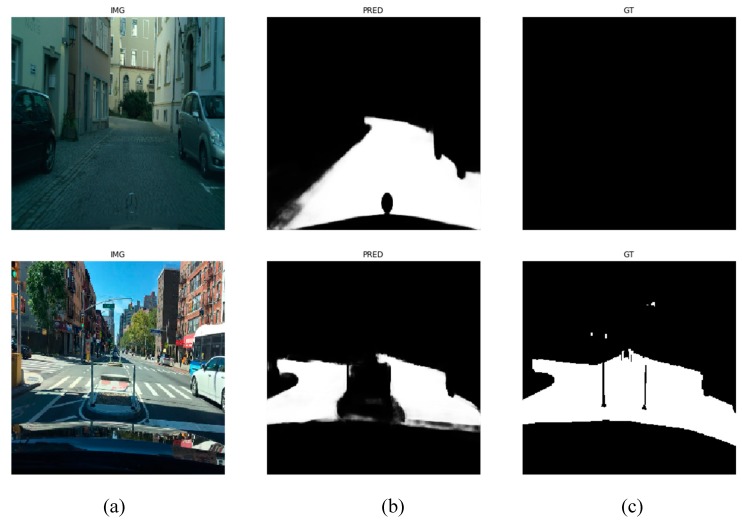
Examples of wrong annotations in the CityScapes [24] and Berkeley Deep Drive [25] datasets. Left column, (**a**)—input image, center column, (**b**)—prediction, right column, (**c**)—ground truth images containing errors.

While investigating the low IoU scores from the evaluation, we found that the validation and training sets contain significant labeling errors, as seen in Figure 13. 

The CNN trained by us on multiple datasets is able to correctly segment the input images, even though the ground truth labeling is not always accurate, and these images might affect some of the metrics. Our system is robust enough to be used in a monocular perception system as the first step to create a measurement map of the scene, and it can always be improved by more training.

### 4.3. Calibration Results

Automatic camera calibration of height and pitch and yaw angles is the first step to be taken when a new camera setup is encountered. We performed three tests using different vehicles and cameras of our own, and we also performed tests on the KITTI (Karlsruhe Institute of Technology and Toyota Technological Institute) dataset [4], which includes information about the camera height. The results are presented in Table 2.

**Figure 14:**
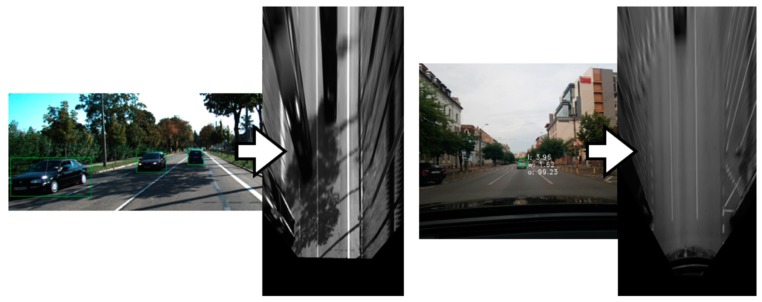
Generating the Inverse Perspective Mapping (IPM) image using the calibration data.

Our test data, and the KITTI dataset, do not include information about the ground truth pitch and yaw angle. Therefore, the only way to assess that computed pitch or yaw angles are correct is to construct the projection matrix, use it for generating bird’s-eye view images, and analyze the resulted images. A good pitch angle will result in IPM images where the lane lines are parallel, and a good yaw angle results in lanes being vertical while driving on a straight road, as can be seen in Figure 14.

### 4.4. Obstacle Detection Results

The obstacle detection algorithm was also evaluated using the publicly-available KITTI tracking dataset. The results on trips labeled 0005, 0010, 0011, and 0018 are illustrated below in Table 3. We computed the mean absolute error between the 3D location of the object detected by our approach and the ground truth from KITTI dataset. The evaluation is done on the Z-axis only, which represents the distance to the detected obstacle and is highly relevant for depth estimation using monocular approaches. Matching the objects is done using IoU applied to the bounding boxes and we have filtered out objects that appear elongated (our system may detect sidewalks or other types of continuous obstacles). Evaluation is done on different distance intervals and the range of our system is limited to 50 m.

For the same trips we have also computed the detection rate, the results being presented below, in Table 4.

The obstacle detection algorithm was also evaluated using a video sequence from a previous research project, consisting of calibrated pairs of images acquired using a binocular camera setup. The ground truth is created from the tracked objects using stereovision-based algorithms, which are able to detect generic objects, regardless of their type, and measure their 3D position with a high degree of accuracy. Table 5 presents the results of this test.

The grid covers a road width of 24 m, meaning that in some cases the objects on the sides are not detected. The scanning algorithm also induces limitations regarding occluded vehicles: A vehicle that is behind another vehicle will not be seen by our measurement model generation algorithm, while a stereovision based algorithm will see parts of it. The detection rate in the nearest distance interval (0–10 m) is affected by the fact that the contact points between the obstacles and the road are often occluded by the ego-vehicle’s hood. At the other extreme, for larger distances, the expected error of the monocular system is high, meaning that the occupancy probability of the grid is low and therefore the objects are not always detected, especially when they pass in and out of the detection range. The stereovision system has multiple advantages over a monocular vision system: the obstacle features are directly extracted as 3D points, no assumptions about the obstacles touching the road are necessary, and the detection range is determined by the camera parameters alone (focal length and baseline). There is no doubt that when available, stereovision is superior. However, setting up a stereovision system is difficult, as the cameras need to be precisely synchronized and calibrated and, therefore, most off-the-shelf cameras or mobile phones cannot be used for this purpose, and also the skills required for setting such a system up are beyond the level of the average vehicle user.

We have attempted to compare our system with existing detection approaches that are based on a monocular camera and available on mobile devices. Many such solutions have extremely poor performance, or are not maintained or updated, and thus were not suitable for comparison. Table 6 presents the results while running on the same image sequence where we manually counted the number of detected vehicles between our system and the mobile application UGV Driver Assistance [31]. The total number of vehicles in the sequence was 89. While exploring other similar solutions on mobile platforms, we found that the UGV application is one of the best and it is mostly based on a CNN that is able to offer bounding box predictions for vehicles present in the scene.

From Table 6 we may observe that the other approach offers a higher detection rate due to the efficiency of the CNN to produce bounding boxes from images. The main downside is that sometimes it may offer too many predictions, it tends to “overshoot” and produce a lot of false-positive detections. We illustrate some comparison examples in the following figure.

**Figure 15:**
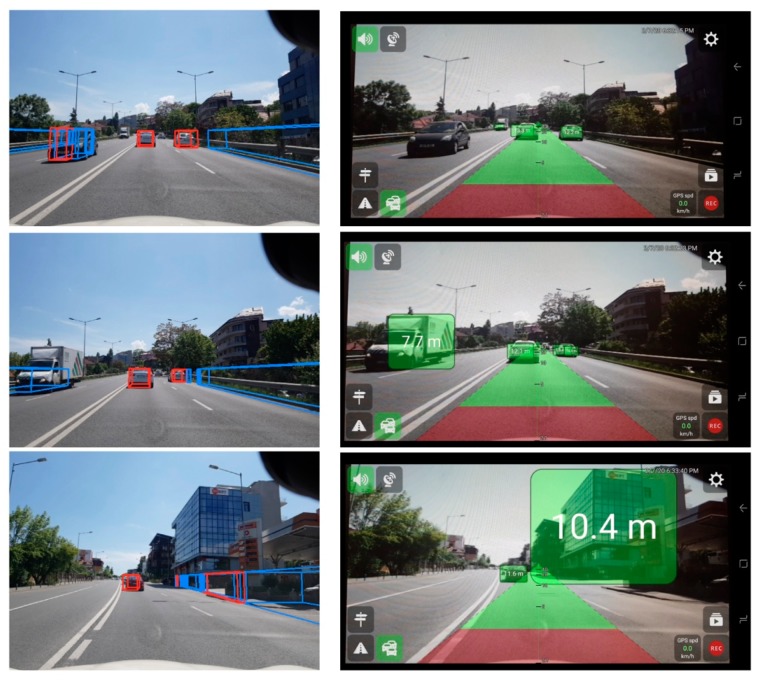
Comparison with a CNN-based detector running on mobile devices. Left column—our approach, right column—UGV Driver Assistant.

From Figure 15 we can observe that, depending on the training data, the CNN might sometimes offer completely false predictions (as can be seen in the last figure of the second column), or it may sometimes even miss detections due to the reduced features (especially darker vehicles, as can be seen in the first figure of the second column). Our proposed solution has a reduced range, due to the limits of the world model, but the detections are robust, especially due to the fact that they are tracked over time.

### 4.5. Running Time

The algorithms are integrated in a C++ application that runs on a generic GPU equipped PC. The application framework can be run on both Windows and Linux operating systems. The neural network used for segmentation was developed and trained using Python, and then successfully exported and integrated in the C++ application.

The entire processing flow, including CNN segmentation, particle creation, and grid state update, and visualization of the results is done in 70–80 ms, depending on the number of objects in the scene. The processing alone, without visualization of intermediate and final results, takes between 40 and 50 ms.

**Figure 16:**
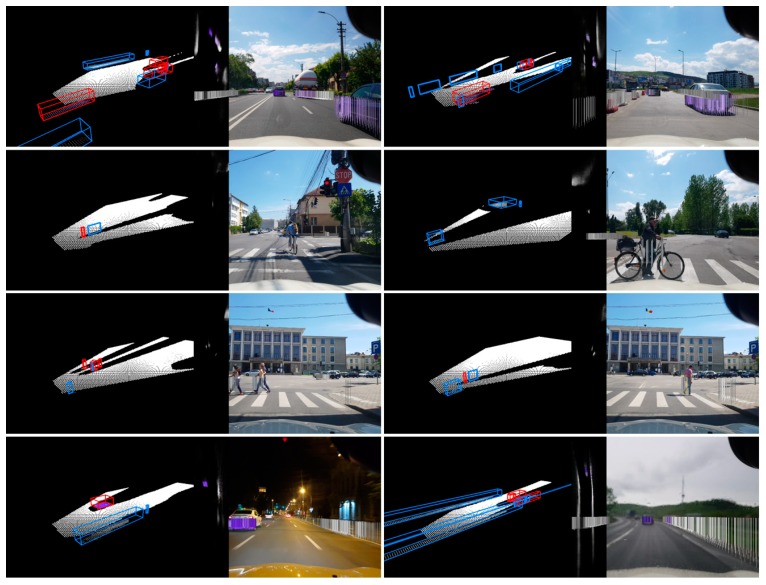
Eight examples with different scenarios from the processing system, each displaying the extracted cuboids and the segmented road surface (left), the color-coded particles in the top-down image view (center) and the particles projected in the color input image (right). Top row—detecting moving vehicles; second row—detecting bicycles; third row—detecting pedestrians; bottom row—detecting obstacles at night (left) or in the rain (right).

Screenshots of the application are shown in Figure 16. The system is able to detect cars, pedestrians, bicyclists, and even continuous structures like fences, in multiple scenarios and weather conditions.

A video of the system running on our own sequences in various scenarios is available at [28].

### 4.6. Comparison with Other Obstacle Detection Techniques

In Table 7 we present a comparison of our system with other state-of-the-art object detection methods, based on features and capabilities.

The first two methods presented in Table 7, described in [9] and [11], are based on convolutional neural networks. Their main limitation is the reliance on obstacle classes for the extraction of 3D information and orientation and, therefore, they are limited to detecting obstacle categories as seen by the network during training. The method described in [32] uses stereovision along with CNNs to generate 3D object proposals, in the form of oriented cuboids, after the scene is processed at voxel level. The method presented in [23] is based on stereo-vision cameras and dynamic occupancy grids, being one of the main ideas behind the results described in the current paper. While the stereovision brings useful 3D information, allowing for a higher detection range and accuracy, it requires very precise, offline calibration, and does not integrate easily other information sources.

The method described in [33] is based on lidar sensor data. The 3D point cloud representation is converted into a 2D representation, which is then analyzed using a fully-connected CNN to generate the oriented bounding boxes of the obstacles. This method is able to achieve generic obstacle detection, and to integrate multiple 3D point data sources as input. Still, using the lidar requires calibration, and also brings the other specific disadvantages of the sensor, such as the need of professional mounting on the vehicle’s exterior.

The solution from [21] employs a CNN for candidate region extraction, then uses another CNN for orientation and size estimation and, in the end, makes use of the LSTM neural network to track the detections. The main drawback is that it heavily relies on training data, the authors even mention that they used extensive synthetic images during the training and development of the approach. The approach is limited to the obstacle types learned by the detection CNN; therefore, it is not generic. The solution does handle vehicle occlusions well.

The method presented by us in this paper is based on a single camera, but has the advantage that it can self-calibrate during normal driving (given a sufficient number of frames), it can detect the speed and orientation of the detected obstacles, and it supports any type of obstacle on the road surface, meaning that it is a generic obstacle detection. The grid representation also has the advantage that it can support multiple sensorial data, meaning that it can easily be extended by adding multiple cameras.

The work presented in this paper represents a generic obstacle-tracking framework based on a monocular camera. A numeric comparison with other state-of-the-art monocular vision methods on publicly-available databases is difficult, because our system requires continuous video sequences, with timestamps and speed and yaw rate data, in order to self-calibrate and then track objects, and these sequences must also have ground truth-detected objects. The 2D detection and evaluation from the popular KITTI framework cannot be used in our case in order to compare with other methods. Additionally, valuation on the KITTI 3D object detection dataset is also not achievable at this moment, due to the fact that the evaluation is done on random images, whereas our system requires images from the same sequence in order to initialize the particle filter and to perform the tracking and then the 3D extraction. Furthermore, the 3D object detection dataset uses three classes: car, pedestrian and cyclists. Whereas the work in this paper proposes a more generic framework that is able to detect any kind of obstacle on the road surface and is not limited to just three classes. We intend to add basic classification in future work.

Running the evaluation on the KITTI 3D vehicle tracking set is also not feasible due to the fact that the dataset is oriented towards the performance of identification of objects as unique persistent entities across frames, and not towards 3D measurement, while our approach does not assign individual tracking IDs to each tracked obstacle, but measures its position, size, and speed. We do, however, plan to address this issue in the near future.

## 5. Conclusions

We have presented a complete solution for the perception of generic obstacles on the road using monocular vision, which combines the segmentation power of the convolutional neural networks with the dynamic environment description power of the occupancy grids. The main contribution of this paper is the description of the steps required to generate a useful probabilistic sensor model for the monocular camera, so that the segmented information from the image space can be used in the world’s space as approximated by the occupancy grid. Additionally, because camera calibration is both essential for establishing the relation between the 3D world and the image space, and also a step that most users of a vision system will gladly avoid, we proposed an automatic calibration technique that does not need user assistance, and only assumes that the user will mount the camera facing in the general direction of the vehicle’s traveling direction.

Combining CNN-based segmentation, occupancy grid tracking, and automatic calibration, we achieved a real-time vision system that works on most traffic scenarios, is able to detect and measure the obstacles within the reasonable accuracy limitations expected from a monocular vision system, and which can be extended to support multiple cameras or ranging sensors such as laser or radar.

## Figures and Tables

**Table 1:** Evaluating segmentation with state-of-the-art, using the Intersect Over Union (IoU) performance metric.

CNN Model	IoU score (road class only)
DeepLab [29]	0.986
E-Net [30]	0.974
Proposed CNN-trained multi-class	0.922
Proposed CNN-trained single-class (road only)	0.911

**Table 2:** Self-calibration results for camera height and pitch angle computation.

Scenario	Estimated Camera Height (mm)	Ground Truth Camera Height (mm)	Pitch Angle (degrees)
Own setup test 1	1268	1250	−1.68°
Own setup test 2	1234	1200	−3.8°
Own setup test 3	1468	1480	2.97°
KITTI setup	1649	1650	−0.85°

**Table 3:** Mean Absolute Error (MAE) analysis on trips from KITTI dataset.

Distance Range (m)	KITTI 0005 MAE (m)	KITTI 0010 MAE (m)	KITTI 0011 MAE (m)	KITTI 0018 MAE (m)
0–10	-	-	0.8	-
10–20	1.30	2.6	3.29	3.91
20–30	3.65	2.74	5.59	5.59
30–40	2.07	6.03	11.72	6.2
40–50	2.08	4.35	19.06	9.32

**Table 4:** Detection rate evaluation on trips from KITTI dataset.

Distance Range (m)	KITTI 0005 Detection Rate (%)	KITTI 0010 Detection Rate (%)	KITTI 0011 Detection Rate (%)	KITTI 0018 Detection Rate (%)
0–10	-	-	97.56	-
10–20	100	94.73	97.25	79.2
20–30	89.62	62.78	82.95	74.4
30–40	76.95	51.85	40.73	74.71
40–50	60	5.35	31.46	47.05

**Table 5:** MAE and detection rate compared with a stereo-vision-based sequence.

Distance Range (m)	Mean Absolute Error (m)	Detection Rate (%)
0–10	0.78	88.66
10–20	1.37	96.03
20–30	2.62	92.32
30–40	7.21	80.61
40–50	17.44	57.43

**Table 6:** Detection analysis compared with a commercial mobile app.

	Number of Vehicles Detected	Detection Rate (%)	Number of False Positive Detections	False Discovery Rate (%)
Ours	88	92.6	5	0.05
UGV [31]	87	91.5	49	34.0

**Table 7:** Feature comparison with state-of-the-art obstacle detection techniques.

Method	Sensor	Self-Calibration	Detect Speed	Detect Orientation	Support Multiple Sensors	Generic Obstacle Detection
Mono 3D [9]	Monocular	No	No	Yes	No	No
OFT-Net [11]	Monocular	No	No	Yes	No	No
3DOP [32]	Stereovision	No	No	Yes	No	No
Danescu et al. [23]	Stereovision	No	Yes	Yes	Yes	Yes
Li et al. [33]	Lidar	No	No	Yes	Yes	No
Hu et al. [21]	Monocular	No	No	Yes	No	No
**Ours**	**Monocular**	**Yes**	**Yes**	**Yes**	**Yes**	**Yes**
